# Transcription Factor ZNF683 Inhibits SIV/HIV Replication through Regulating IFNγ Secretion of CD8+ T Cells

**DOI:** 10.3390/v14040719

**Published:** 2022-03-30

**Authors:** Ying Lu, Ming-Xu Zhang, Wei Pang, Tian-Zhang Song, Hong-Yi Zheng, Ren-Rong Tian, Yong-Tang Zheng

**Affiliations:** 1 Key Laboratory of Animal Models and Human Disease Mechanisms of the Chinese Academy of Sciences, Kunming Institute of Zoology, Chinese Academy of Sciences, Kunming 650223, China; luying@mail.kiz.ac.cn (Y.L.); mingxu2008-2009@163.com (M.-X.Z.); pangw@mail.kiz.ac.cn (W.P.); songtianzhang@mail.kiz.ac.cn (T.-Z.S.); zhenghongyi@mail.kiz.ac.cn (H.-Y.Z.); tianrenrong@mail.kiz.ac.cn (R.-R.T.); 2 Kunming College of Life Science, University of Chinese Academy of Sciences, Kunming 650204, China

**Keywords:** lung, SIVmac239, slow progressors, rapid progressors, northern pig-tailed macaques, ZNF683

## Abstract

Pulmonary microbial invasion frequently occurs during AIDS progression in HIV patients. Inflammatory cytokines and other immunoregulatory factors play important roles in this process. We previously established an AIDS model of SIVmac239 infection in northern pig-tailed macaques (NPMs), which were divided into rapid progressor (RP) and slow progressor (SP) groups according to their AIDS progression rates. In this study, we performed 16S rDNA and transcriptome sequencing of the lungs to reveal the molecular mechanism underlying the difference in progression rate between the RPs and SPs. We found that microbial invasion in the RP group was distinct from that in the SP group, showing marker flora of the *Family XI*, *Enterococcus* and *Ezakiella*, and more *Lactobacilli*. Through pulmonary transcriptome analysis, we found that the transcription factor ZNF683 had higher expression in the SP group than in the RP group. In subsequent functional experiments, we found that ZNF683 increased the proliferation and IFNγ secretion ability of CD8+ T cells, thus decreasing SIV or HIV replication, which may be related to AIDS progression in SIVmac239-infected NPMs. This study helps elucidate the various complexities of disease progression in HIV-1-infected individuals.

## 1. Introduction

HIV infection remains a global health concern. Although highly active antiretroviral therapy can prolong the lifespan and improve the quality of life of patients [1], there is no effective way to eliminate HIV completely [2]. Infection with HIV leads to a decrease in CD4+ T cells and ultimately develops into AIDS in patients who do not receive highly active antiretroviral therapy [3]. Some patients are infected with the virus but show little change in lymphocyte numbers; these patients are known as elite controllers or long-term non-progressors (LTNPs) [4]. The mechanism of virus control in LTNPs has not been well studied. Broadly neutralizing antibodies play a major role in the antiviral process [5,6,7], as does cellular immunity. Virus-specific CD8+ T cells mediate the cytotoxic T lymphocyte (CTL) response more strongly in LTNPs than in rapid progressors (RPs) [8,9]. The function of immune cells is associated with the disease progression of HIV. Notably, the molecular mechanism of virus control by LTNPs has become a popular research topic that is promoting the entry of new techniques, such as multiple omics analyses, into the field.

In addition to CD4+ T cell decline and high levels of secondary viremia, another characteristic of AIDS is microbial invasion with the collapse of immunity, which can easily lead to a strong inflammatory response, thus promoting the replication of HIV and accelerating the progression of disease [10]. The respiratory mucosa directly contacts the external environment through breathing, which increases the probability of infection with a pathogen. Despite significant progress in treatment, bacterial pneumonia still has a significant impact on mortality in HIV-infected patients, especially among people who lack medical services, who often suffer from acute opportunistic infections associated with AIDS [11,12,13,14].

The mucosal immune system is the main site of HIV infection, and the lungs are important components of the mucosal immune system and effector sites of HIV replication [15]. One study on rhesus monkeys infected with simian immunodeficiency virus (SIV) showed that the immune responses in the lungs were significantly different from those in peripheral blood [15]. The immune cells in the lungs activate and produce large numbers of cytokines, mainly IFNγ and IFNα, to resist infection [16].

Animal models are very important in the study of HIV infection [17,18]. Nonhuman primates are similar to humans in many ways, which makes them the most suitable animal model to study HIV infection [19]. SIV infections in macaques result in an AIDS-like disease [20,21]. Our previous study found that northern pig-tailed macaques (NPMs) infected with SIVmac239 could be grouped into two different disease progression phenotypes: slow progressors (SPs) and RPs. In the SP group, the viral load in blood was low, the number of CD4+ T cells showed no obvious change, and there was no AIDS-related disease [22,23]. This macaque model is a valuable resource for understanding AIDS progression. In this study, using microbiome and transcriptome sequencing, we profiled the pulmonary microbial compositions and gene changes in the two groups of SIVmac239-infected NPMs. In subsequent cellular assays, we identified a transcription factor, ZNF683, that was highly expressed in the lung tissues of the SP group and was detected at similarly high levels in HIV patients. ZNF683 increased the proliferation and IFNγ secretion ability of CD8+ T cells, and when it was knocked down, HIV replication was increased. This gene may be related to CD8+ T cell function and AIDS progression in SIVmac239-infected NPMs and may be a new diagnostic or therapeutic marker in HIV patients.

## 2. Materials and Methods

### 2.1. Sample Collection

The animals used in this experiment, which included 10 NPMs, were checked and confirmed to be free of simian T-lymphotropic virus, simian retrovirus type D, SIV, and simian cytomegalovirus, as mentioned in previous studies [23]. Protocols related to feeding, sampling and anesthesia throughout the experiment were carried out in accordance with the requirements of the Kunming Institute of Animal Research ethics committee under approval number SYDW-2015023. For virus infection, each macaque was intravenously injected with 3000 50% tissue culture infectious dose (TCID50) units of the SIVmac239 strain [23]. Ketamine hydrochloride was used to anesthetize the animals. Lung tissues were obtained at necropsy and immediately stored at −70 °C or soaked in paraformaldehyde. A survey was conducted among HIV-1-infected individuals at Cangyuan Wa Autonomous County People’s Hospital. All participants provided written informed consent. The original study protocol was reviewed and approved by the Ethics Committee of Kunming Institute of Zoology, Chinese Academy of Sciences (approval number: SWYX-2013023). Blood samples were collected into EDTA Vacutainer tubes by venipuncture, and peripheral blood mononuclear cells (PBMCs) were separated by Ficoll.

### 2.2. Viral Loads

Approximately 1 mg of lung tissue was collected and homogenized with a tissue cell destroyer (NZK, Wuhan, China). Total RNA was extracted with RNAiso Plus (TaKaRa, Dalian, China) and quantified with a NanoDrop 2000 (Thermo Scientific, Waltham, MA, USA). Approximately 1 μg of lung RNA was prepared for viral RNA detection, and viral RNA was extracted from the cell culture supernatant with a High Pure Viral RNA Kit (Roche Diagnostics GmbH, Roche Applied Science, Mannheim, Germany) according to the manufacturer’s instructions. The viral loads in tissues and supernatant were determined by quantitative PCR (TaqMan) as described in our previous reports [23,24].

### 2.3. Immunofluorescence Assay

An immunofluorescence assay was performed as described previously [25]. Briefly, slices soaked in sodium citrate buffer were heated in a microwave for 6 min three times for antigen retrieval. Then, the slices were cooled to room temperature, washed with 1× PBS containing 0.05% Tween-20 (1× PBST), and incubated with 0.1% Triton X-100 at room temperature for 10 min. The sections were then washed, treated with 3% hydrogen peroxide at room temperature for 10 min, washed again, and sealed with 10% bovine serum albumin at 37 °C for 60 min. The primary antibody was diluted in bovine serum, and the samples were cultured with the antibody at 4 °C overnight or at 37 °C for 60 min. Then, the slices were washed and incubated with the secondary antibody in BSA for 60 min at room temperature. The primary antibodies used were a rabbit monoclonal anti-CD4 antibody (ab133616, Abcam, Cambridgeshire, United Kingdom, 1:1000) and a rabbit polyclonal anti-CD8 antibody (ab4055, Abcam, Cambridgeshire, United Kingdom, 1:400), and the secondary antibodies were goat anti-rabbit (Alexa Fluor 555, ab150078, Abcam, Cambridgeshire, United Kingdom, 1:2000) and goat anti-rabbit (Alexa Fluor 488, ab150077, Abcam, Cambridgeshire, United Kingdom, 1:3000) antibodies. Finally, a Leica DMI4000B Microsystems microscope (Leica Microsystems, Wetzlar, Germany) was used for observation.

### 2.4. Microbiome Sequencing

Lung DNA was extracted using a QIAamp stool DNA kit (Qiagen, Hilden, Germany). The DNA concentration and purity were measured using a NanoDrop 2000 spectrophotometer (Thermo Scientific, Waltham, MA, USA). The 16S rDNA of the lungs was sequenced by Biomarker Technologies Corporation. To avoid the influence of long sequences on sequencing, the general primer pair 515F/806R was used to amplify the bacterial 16S rDNA sequence. The primer sequences were 5′-GTGYCAGCMGCCGCGGTAA-3′ (515F) and 5′-GGACTACHVGGGTWTCTAAT-3′ (806R). Each sample was tested in triplicate, and the PCR products were detected by agarose gel electrophoresis. The concentration and specificity were qualified for subsequent high-throughput sequencing. The 16S rDNA of the lungs was sequenced using the Illumina HiSeq platform. The top 20 flora in the lungs in terms of relative abundance were analyzed, and linear discriminant analysis (LDA) effect size (LEfSe) analysis was used to identify biomarker microorganisms with significant differences between the groups. The threshold for LDA was 4. The data were analyzed using the open-source software Biomarker BMKCloud (https://console.biocloud.net, (accessed on 3 January 2021)). All the sequence data were submitted to the Sequence Read Archive (SRA) database, and the accession numbers were SRR9278630 to SRR9278631, SRR9278634 to SRR9278639, and SRR9278643 to SRR9278644. The samples were divided into three groups: the SP group (N = 3); the RP group (N = 3); and the healthy group (N = 4).

### 2.5. Histological Examination

Tissues were fixed in 4% paraformaldehyde, dehydrated in a graded ethanol series, and embedded in paraffin. The paraffinized tissue sections were then deparaffinized, rehydrated, and stained using the hematoxylin-eosin (HE) method [26].

### 2.6. Transcriptome Sequencing

Total RNA from lung tissues was extracted using TRIzol reagent (Invitrogen, Carlsbad, CA, USA), and the RNA was reverse-transcribed into cDNA using a PrimeScript^TM^ RT Reagent Kit (TaKaRa) according to the manufacturer’s instructions. A cDNA library was established using an RNA-Seq Library Preparation Kit (Illumina, CA, USA) following the manufacturer’s protocol and used for Illumina HiSeq sequencing. FastQC software was used for quality control of the original data, and Trimmomatic software (Illumina, CA, USA) was used to preprocess the original data to remove the ribosomal RNA sequences and other possible interfering contaminants. The clean reads were aligned to a reference genome (https://www.ncbi.nlm.nih.gov/genome/?term=Macaque+nemestrina, (accessed on 1 March 2018)). All the sequence data were submitted to the SRA database, and the accession numbers were SRR9050947 to SRR9050956. The criteria for differentially expressed genes (DEGs) were a false discovery rate (FDR) of 0.01 and a log2 (fold change) ≥ 2. The significantly enriched pathways of the DEGs were determined by using Kyoto Encyclopedia of Genes and Genomes (KEGG) pathway analysis, which was performed using KOBAS software. STRING online software was used to search for the protein–protein interaction (PPI) network of the proteins encoded by the DEGs [27].

### 2.7. Cytokine Gene Expression Assay

Total RNA isolated from tissues and PBMCs was reverse-transcribed into cDNA with a PrimeScript^TM^ RT Reagent Kit with gDNA Eraser (Takara). Real-time qPCR was performed on a ViiA7 Real-Time PCR System using SYBR Premix Ex Taq II (Takara). The expression levels of target genes were analyzed using the comparative cycle threshold (Ct) method, where Ct was the cycle threshold number normalized to that of GAPDH mRNA or 18S mRNA, according to a previous study [24]. The sequences of the related primers are shown in Table 1.

### 2.8. Flow Cytometry

To detect the secretion of IFNγ, PBMCs were treated with phorbol myristate acetate (PMA), monensin, and brefeldin-A (BFA) for 6 h. Then, flow cytometry was performed as previously described [28]. For surface staining, after washing with staining buffer (PBS with 2% newborn calf serum and 0.09% sodium azide), the residual cells were resuspended in staining buffer containing the relevant monoclonal antibodies (mAbs) for 30 min on ice. For IFNγ staining, surface-labeled cells were further treated with fixation and permeabilization solution (BD Biosciences, San Jose, CA, USA) followed by perm/wash buffer (BD Biosciences, San Jose, CA, USA) and then stained using antibodies on ice for 30 min. Anti-human mAbs that cross-reacted with macaques were used according to standard procedures. Anti-CD8 PE-Cy7 (clone RPA-T8, 557746), anti-CD3 APC-Cy7 (clone SP34-2, 557757), anti-CD4 FITC (clone OKT4, 566802), and anti-IFNγ-PE (clone 4S.B3, 559326) were purchased from BD Pharmingen (Franklin Lakes, NJ, USA).

For carboxyfluorescein succinimidyl ester (CFSE) detection, fresh PBMCs were cultured overnight in medium containing IL-2. On the second day, a cell suspension was made and incubated with 10 μM CFSE (Cayman, MI, USA) solution at 37 °C for 10 min. The staining was terminated with 10% fetal bovine serum (Gibco, MA, USA), and the cells were washed 3 times. The experimental group was stimulated with concanavalin A (Cayman) or phytohemagglutinin (Sigma, St. Louis, MO, USA), while the control group was not. After 72 h of culture, the signal intensity of CFSE was detected.

### 2.9. Cells

PBMCs were maintained in RPMI 1640 (Gibco) containing 10% heat-inactivated fetal bovine serum and IL-2 (50 U/mL; Biotest, Germany). The human PBMCs and CD8+ T cells were transduced using Lipofectamine^TM^ 2000 with a control sequence (Santa Cruz Biotechnology, sc-37007, Santa Cruz, CA, USA) or *ZNF683* siRNA (Santa Cruz Biotechnology, sc88560) according to the manufacturer’s instructions. NPM PBMCs with ZNF683 knockout were made with Cas9X^TM^ by HaiXing Biotechnology Company (Changsha, China) Total CD8+ T and CD4+ T and natural killer (NK) cells were isolated using magnetic sorting with a CD8+ T cell isolation kit, CD4+ T cell isolation kit and NK cell isolation kit (Miltenyi Biotec, Bergisch Gladbach, Germany).

### 2.10. Virus Strain and Infection In Vitro

The HIV-1_NL4−R3A_ provirus plasmid was donated by Prof. Liguo Zhang (Institute of Bio physics, Chinese Academy of Sciences). The HIV-1_NL4−R3A_ strain was produced in 293T cells (Type Culture Collection of the Chinese Academy of Sciences, TCCCAS) by transfecting the provirus plasmids using Lipofectamine^TM^ 2000 according to the manufacturer’s instructions (Invitrogen, Waltham, MA, USA). Viruses were harvested 48 h post-transfection, and 1 mL aliquots of the virus-containing supernatants were frozen at −80 °C until use. A total of 1 × 10^5^ PBMCs from healthy donors were infected with virus at a multiplicity of infection (MOI) of 0.01, and replication was monitored by quantitative PCR of the supernatant at 3-day intervals post-infection.

### 2.11. Western Blotting Assay 

The cells were lysed using lysis buffer (Beyotime, Shanghai, China) on ice for 10 min. Protein extract was then loaded on a 10% polyacrylamide gel and electroblotted onto a polyvinylidene fluoride (PVDF) membrane and blocked for 2 h. The membrane was then incubated with GAPDH (Abcam, Cambridgeshire, United Kingdom, ab9485) and ZNF683 (Invitrogen, PA1-30046), antibodies at 4 °C overnight. After washing with TBST, corresponding HRP-labeled secondary antibodies were added and incubated at room temperature for 1 h, then washed again using TBST. Bands were visualized using an ECL western blotting system (Tanon, Shanghai, China).

### 2.12. Luciferase Assay

We cloned the promoter region of IFNγ on pGL3-basic vector and *ZNF683* on pcDNA3 1 vector. 293 cells were co-transfected with 500 ng pGL3-IFNγ- promoter, 1μg pcDNA3.1-ZNF683, and 100 ng pRenilla. After 48 h, the luciferase activity was detected by Dual-Luciferase^®^ Reporter Assay System (Promega, Madison, WI, USA).

### 2.13. Statistical Analysis

Statistical analyses were performed with GraphPad 8.0.1 (GraphPad Software, San Diego, CA, USA), and paired *t* tests were used to compare data. The data are presented as the means ± SEMs. *p* < 0.05 was regarded to indicate statistical significance.

## 3. Results

### 3.1. Low Levels of Viral Replication and Superior CD4+ T Cell Homeostasis in Lung Tissues Were Found in the SIVmac239-Infected SP Group

**Figure 1:**
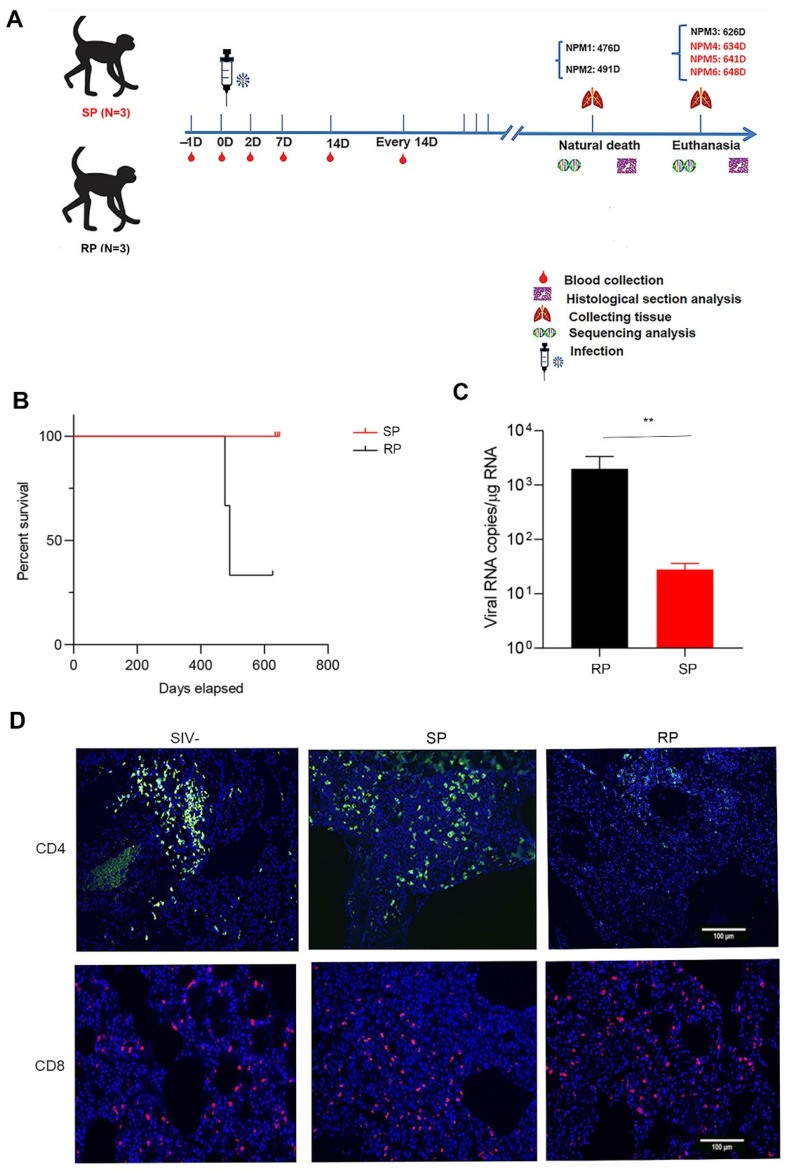
Viral load and changes in T cells in the lungs during chronic infection. (**A**) Schematic of the study. Blood samples were collected twice in the first week after infection, once in the second week after infection, and once every 14 days for the remainder of the time. The animals that died naturally were dissected immediately, and lung tissue was collected. The other animals were euthanized. SP: *n* = 3, RP: *n* = 3. (**B**) Survival curves of the two groups. (**C**) Viral load in the lungs. (**D**) Changes in T lymphocytes in the lungs before and after infection with SIVmac239 (blue: DAPI; green: CD4; red: CD8). Representative figures were selected randomly. Slow progressors/SP: *n* = 3, Rapid progressors/RP: *n* = 3, Healthy/SIV-: *n* = 4. The bars and error bars indicate the means ± SEMs. Paired t tests were used to compare groups. ** *p* < 0.01.

We inoculated each NPM intravenously with 3000 TCID50 of the SIVmac239 strain (Figure 1A). The SIVmac239-infected NPMs could be divided into slow progressors (SP) and rapid progressors (RP) groups according to disease progression (Figure 1B). The virological and immunological characteristics of peripheral blood were described in previous study [22]. As pulmonary infections are common outcomes and are associated with the development of AIDS, we explored the lung-related virological and immunological parameters of the two groups after SIVmac239 infection. First, we monitored pulmonary-associated viral RNA, which was rarely detected in the SP group and present at much lower levels in the SP group than in the RP group (Figure 1C). Furthermore, we monitored the changes in T cells in lung tissues. Compared with the RP group, the SP group showed more CD4+ T cell homeostasis, which was consistent with the condition in the peripheral blood (Figure 1D). In addition, there was no significant difference in the reduced ratio of CD8+ T cells between the two groups (Figure 1D).

### 3.2. Microbial Invasion Was Significant in the Lungs of RPs

**Figure 2:**
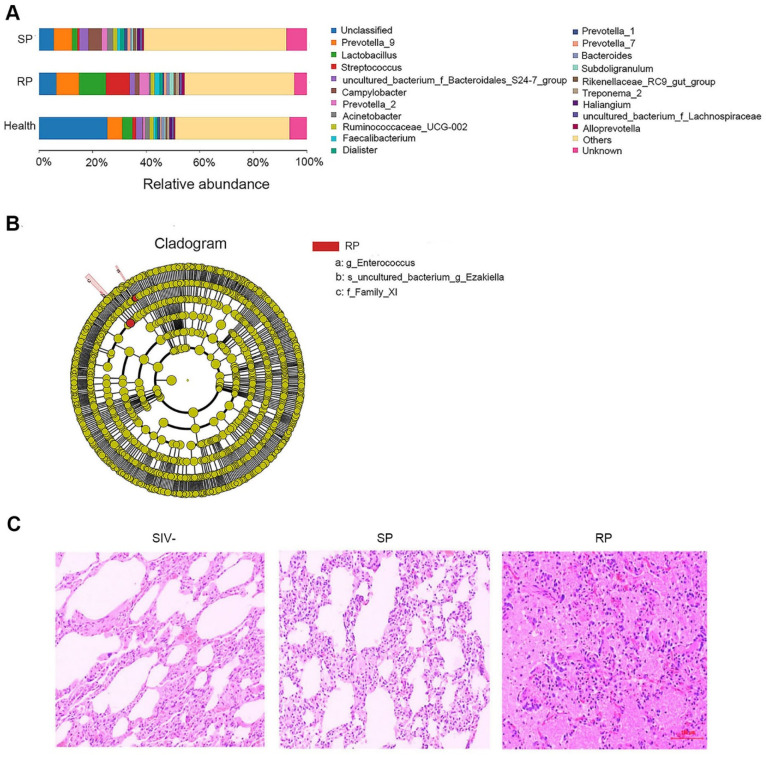
Changes in microbial flora in the lungs during primary infection with SIVmac239. (**A**) The relative abundance of flora in the lungs of the SP, RP, and healthy groups. The bar graph shows only the top 20 species at the abundance level and merges other species into “Others”. “Unknown” represents species that have not been taxonomically annotated, and the different colors represent different flora. (**B**) Cladogram from LEfSe analysis. The circles radiating from the inside to the outside represent the classification levels of the microbes from phylum to species (g: genus; s: species; f: family); each small circle at the different classification levels represents a classification at this level, and the diameter of the small circle is proportional to the relative abundance. Species without significant differences are uniformly colored yellow, and other species with significant differences are colored red. (**C**) HE staining of lungs. Representative figures were selected randomly. SP: *n* = 3, RP: *n* = 3, SIV-: *n* = 4.

The lungs are similar to the intestines and have a rich microbial community. After HIV infection, microbial invasion often occurs in the lungs. We extracted the total RNA of lung tissue in the two groups of SIV-infected macaques and sequenced the 16S rDNA. The results showed that the microbial compositions of the two groups were different from that of the healthy group; for example, the amounts of *Streptococcus* and *Lactobacillus* in the RP group were increased significantly (Figure 2A). In addition, LEfSe analysis showed that the abundances of *Family XI*, *Enterococcus* and *Ezakiella* bacteria in the RP group were distinct from those in the other two groups and that these microbes are potential marker microorganisms for RPs among SIV-infected NPMs (Figure 2B and Figure S1).

Generally, pathological lesions occur concomitantly with microbe invasion and inflammation [14]. Therefore, we assessed pathological damage in the lungs of RP and SP groups after infection by HE staining. Both pulmonary structures of SIV-infected RP and SP groups showed some pathological lesions. However, the lungs of SPs displayed only pulmonary interstitial thickening (Figure 2C), while the lungs of RPs exhibited obvious bleeding, edema, and more serious lesions (Figure 2C). More representative pictures were shown in Figure S2.

### 3.3. KEGG Pathway Enrichment Analysis of the DEGs

**Figure 3:**
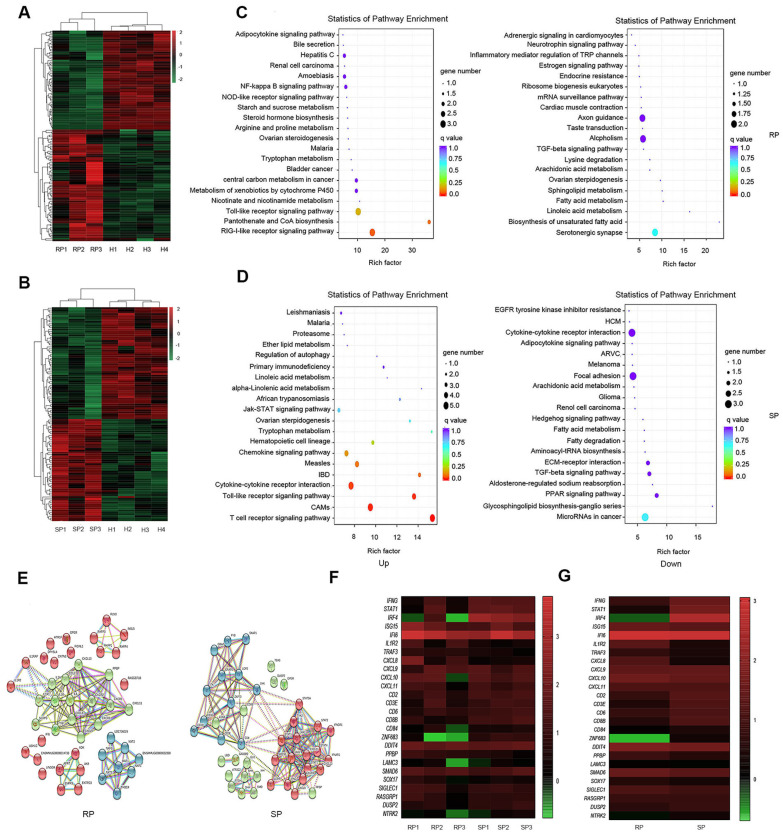
Transcriptomic characteristics in the lungs of the SP and RP groups after infection. Heatmap of DEGs after infection in the RPs (**A**) and SPs (**B**). Scatter diagrams of the enriched metabolic pathways of the DEGs (gene with up- and downregulated expression) are shown for the RPs (**C**) and SPs (**D**). Each dot represents a metabolic pathway, the ordinate shows the name of the pathway, and the abscissa shows the enrichment factor, indicating the proportion of DEGs annotated to the pathway compared to the proportion of all genes annotated to the pathway. (**E**) PPI network of the DEGs during primary infection with SIVmac239 in the RPs and SPs. The nodes represent proteins, and the edges represent protein–protein interactions. Heatmaps of several DEGs in every infected sample (**F**) and the two groups (**G**) are shown. The color represents the expression level: from green to red indicates that the expression level is from low to high. SP: *n* = 3, RP: *n* = 3, SIV-: *n* = 4.

To further determine the reasons for the stability of pulmonary microorganisms in the lungs of SP group, we performed transcriptome sequencing of infected and healthy lung tissues. Based on pulmonary RNA samples from 4 healthy NPMs, 3 RPs, and 3 SPs, we established 10 cDNA libraries to carry out transcriptome analysis. We compared the gene expression of the SP group and the RP group with that of the healthy group to identify the differentially expressed genes (DEGs). By screening the DEGs, we found that there were 53 upregulated genes and 58 downregulated genes in the SP group and 69 upregulated genes and 61 downregulated genes in the RP group compared with the healthy group (Figure 3A,B and Figure S3).

Then, we conducted signaling pathway enrichment analysis. The results showed the 20 pathways with the lowest Q-values in both the SP and RP groups compared with the healthy group and revealed that the main differences between the two groups in terms of the upregulated signaling pathways may involve T cell activation, inflammatory responses, and interferon responses. In the SP group, T cell activation signaling pathways such as the T cell receptor signaling pathway and interferon-related signaling pathways such as the Jak-STAT signaling pathway were upregulated. In RPs, inflammatory response signaling pathways, such as the NF-kappa B signaling pathway, were enhanced (Figure 3C,D).

We also constructed a PPI network using STRING software (Figure 3E) to explore the interaction of these DEGs after infection. As expected, the PPI network of the DEGs in the RP group was mainly associated with the inflammatory response, which was represented by NLRP3. In the SP group, the PPI network of the DEGs was divided into two parts: interferon-related DEGs, which were represented by Jak, STAT, and IFNγ; CD3E and ZAP70, which were related to T cell activation (Figure 3E).

We compared the immune-related DEGs between SPs and RPs. The results showed that interferon-related genes, such as STAT1, IRF4, and IFNγ, and genes related to immune cell function, such as CD8B and CD3E, were upregulated in the SP group. Inflammation-related genes, such as TRAF3 and IL1R2, were upregulated in the RP group. In addition, we were surprised to find that ZNF683 had significant differences between the two groups (Figure 3F,G).

ZNF683 is a transcription factor that can enhance immunity by immediately promoting lymphocyte activation. It is a homolog of blimp-1 in T cells that mediates a transcriptional program in various innate and adaptive immune cell types, such as tissue-resident memory T (Trm) and natural killer T (NKT) cells. Human ZNF683 was uniformly expressed in effector-type CD8+ T cells but not in naive CD8+ T cells or in most memory CD8+ T cells and was strongly correlated with T-bet and IFNγ expression within the CD8+ T cell population. Notably, ZNF683 is necessary for IFNγ production after human cytomegalovirus infection [29]. However, its role and correlation with HIV infection have not been studied [30].

### 3.4. Gene Expression Confirmation by Real-Time PCR

**Figure 4:**
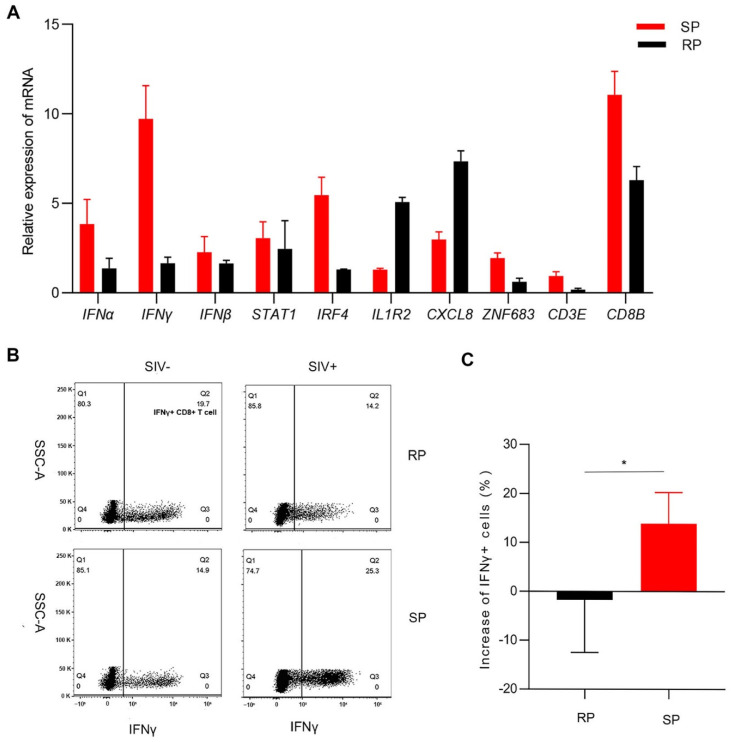
Confirmation of the expression of several DEGs. (**A**) Determination of *DEGs* expression by real-time PCR in the lungs. Changes (**B**) and quantitative analysis (**C**) of IFNγ+ CD8+ T cells after infection in PBMCs. The Q2 gate represents IFNγ+ CD8+ T cells. The bars and error bars indicate the means ± SEMs. Paired t tests were used to compare groups. * *p* < 0.05. RP: *n* = 3; SP: *n* = 3; SIV-: *n* = 4.

Through transcriptome analysis, we found that the signaling pathways of T cell activation and interferon responses were upregulated in the SP group; these pathways play major roles in inhibiting infection. This result is consistent with observations in HIV-1-infected individuals. The inflammatory response signaling pathway was upregulated in the RP group. Therefore, we selected genes with minimal individual differences that were involved in the three biological processes for analysis. We determined the expression of these genes by real-time PCR, and the results were consistent with the transcriptome sequencing results. The expression of ZNF683, IFNγ, CD8B, CD3E, STAT1, and IRF4 was lower in the RP group than in the SP group. However, the expression of IL1R2 and CXCL8 was higher in RPs than in SPs (Figure 4A). CD3E, CD8B, and ZNF683 are all related to T lymphocyte activation and development, especially for CD8+ T lymphocytes. IFNγ is a type II interferon and a common antiviral factor, and it is mainly secreted by lymphocytes. Therefore, we speculated that there may be great differences in the function of CD8+ T cells between the two groups.

We then detected the function of CD8+ T cells in peripheral blood after infection. The proportion of IFNγ+ CD8+ T cells in the SP group was significantly higher than that in the RP group after infection (Figure 4B,C).

Together, these results indicate that the function of CD8+ T cells in SPs may be stronger than that in RPs.

### 3.5. ZNF683 Regulated IFNγ Production and Proliferation of CD8+ T Cells in NPMs and Humans

**Figure 5:**
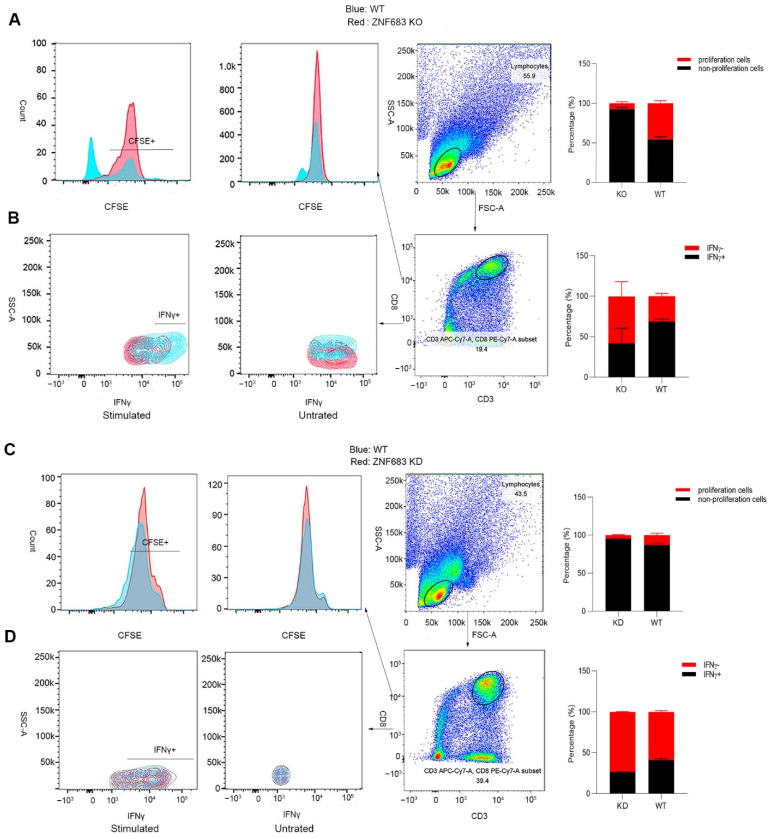
Analysis of the role of ZNF683 in CD8+ T cells in NPMs and humans. (**A**) ZNF683 increased the proliferation of CD8+ T cells in NPMs. (**B**) ZNF683 promoted the production of IFNγ of CD8+ T cells in NPMs. (**C**) ZNF683 increased the proliferation of CD8+ T cells in humans. (**D**) ZNF683 promoted the production of IFNγ of CD8+ T cells in humans. Each experiment was repeated two times, and representative figures were selected randomly.

ZNF683 was sufficient for CD8+ T cells to produce IFNγ, and we also detected elevated expression of ZNF683 in the SP group. Thus, we examined the effect of ZNF683 on CD8+ T cell function and proliferation. When ZNF683 was knocked out in PBMCs from NPMs, the proliferation of CD8+ T cells was inhibited, and the production of IFNγ was reduced (Figure 5A,B). After knockdown of ZNF683 in human PBMCs, the ability of CD8+ T cells to proliferate and secrete IFNγ was also decreased (Figure 5C,D). These results confirm that ZNF683 regulates IFNγ production and proliferation of CD8+ T cells in NPMs and humans.

### 3.6. ZNF683 Was Upregulated in HIV Patients and Inhibited HIV Infection In Vitro

**Figure 6:**
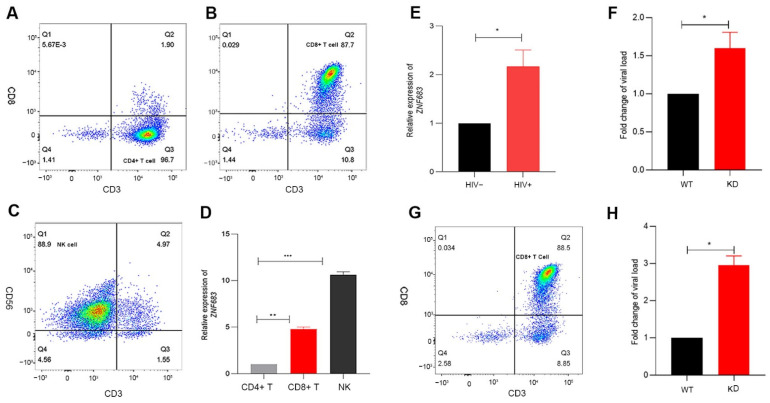
Analysis of the role of ZNF683 in HIV infection. (**A**) Isolation of CD4+ T, (**B**) CD8+ T and (**C**) NK cells from healthy human PBMCs and (**D**) expression of ZNF683 in different human immune cells. (**E**) Expression of ZNF683 in HIV patients. (**F**) ZNF683 inhibited HIV infection in vitro. (**G**) Isolation of CD8+ T cells and (**H**) ZNF683+ CD8+ T cells inhibited HIV infection in vitro. The bars and error bars indicate the means ± SEMs. Paired t tests were used to compare groups. * *p* < 0.05, ** *p* < 0.01, *** *p* < 0.001.

Based on the previous results obtained with the SIV infection animal model, we continued to study the function of ZNF683 in HIV patients. We isolated CD4+ T cells, CD8+ T cells, and NK cells from human PBMCs (Figure 6A–C). Consistent with other reports, ZNF683 was highly expressed in CD8+ T cells in humans (Figure 6D).

We detected the mRNA expression of ZNF683 in HIV patients compared with healthy people and found that ZNF683 was upregulated after HIV infection (Figure 6E). The results of an in vitro infection experiment showed that after knockdown of ZNF683 in human PBMCs, HIV replication increased significantly, as indicated by an increased viral load (Figure 6F). In addition, we isolated CD8+ T cells from healthy human PBMCs (Figure 6G), knocked down ZNF683, and then replenished the isolated CD8+ T cells in the remaining non CD8+ T mixed cells. The knockdown effect of primary cells was detected by Western blot (Figure S4). The treated cells were infected in vitro, and the results showed that after knocking down ZNF683 in CD8+ T cells, the virus replication also increased significantly (Figure 6H). Therefore, we concluded that after HIV infection, ZNF683 expression was upregulated to promote the secretion of IFNγ and the proliferation of CD8+ T cells, resulting in inhibition of viral replication and slowing the disease progression.

**Figure 7:**
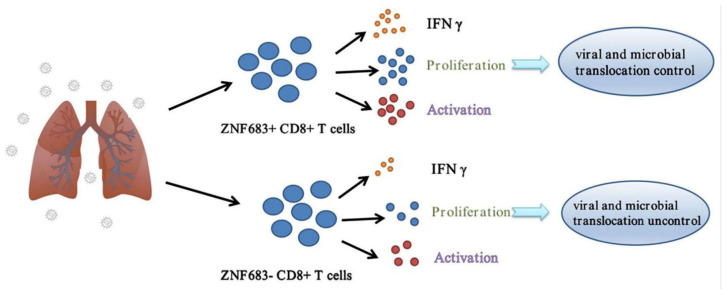
Role of ZNF683 in HIV infection.

Based on our results, NPMs infected with SIVmac239 can be divided into SP and RP groups, and there are significant differences in CD8+ T cell function between the two groups. The important transcription factor ZNF683 promotes the proliferation and IFNγ secretion of CD8+ T cells, thereby inhibiting viral replication and microbial translocation (Figure 7).

## 4. Discussion

Microbial translocation usually means that gastrointestinal microorganisms break through the epithelial barrier and enter the systemic circulation [31]. With the circulation, they can enter many tissues of the whole body. It is the most common phenomenon in AIDS patients, which can cause severe systemic inflammatory response and is one of the signs of disease progression [31,32]. In our experiment, NPMs infected with SIVmac239 were divided into two groups, the RP and SP groups, according to the progression of the disease. To understand the microbial composition of the two groups and examine the correlation with disease progression, we sequenced 16S rDNA from the lungs. LEfSe analysis showed that the levels of *Family XI, Enterococcus,* and *Ezakiella* were significantly different in the RP group than in the SP and healthy groups. Thus, these microbes served as marker microorganisms of RPs. *Enterococcus, family XI,* and *Ezakiella* are all normal intestinal microorganisms [33,34,35]. The increased levels of these microorganisms in the RPs indicated that microbial translocation, and consequent activation of the signaling pathways related to inflammatory response happened in these macaques, which further accelerate the disease process, and is consistent with clinical data in humans [36,37,38]. After HIV infection, an immune response is induced that inhibits viral replication and spread. The inflammatory response is one mechanism for resisting infection. The main inflammatory factors include IL1β, IL6, and TNFα. However, some other studies have stated that activated CD4+ T cells are the target cells of HIV, and the persistent inflammatory response leads to overactivation of the immune system, thus promoting viral replication and accelerating the disease process [39]. In RPs, inflammation-related genes and signaling pathways, such as the TRAF3 and NF-Kappa B signaling pathways, were upregulated after infection. These results are similar to those of other studies and consistent with persistent inflammatory responses associated with disease progression [40].

Interferons have antiviral effects and are mainly divided into type I and type II interferons [41]. Type I interferons are primarily antiviral [42], while type II interferons also have strong immunoregulatory functions that can enhance the biological effects of macrophages, such as the killing of invasive pathogenic microorganisms and intracellular pathogens [43]. After interacting with their receptors, interferons can activate the Jak-STAT signaling pathway and induce the generation of many downstream interferon-stimulated genes (ISGs), which can produce rapid responses to infection, inhibit viral replication and control virus diffusion [44]. In SPs, interferon-producing signaling pathways including the Toll-like receptor signaling pathway and interferon response signaling pathways such as the Jak-STAT signaling pathway were upregulated. The PPI network analysis also showed that the main interferon-related proteins were Jak, STAT1, and IFNγ, while the proportion of the network involved in the inflammatory reaction was small in SPs. In addition, we detected the expression of these genes in the two infection groups by real-time quantitative PCR. The results of our animal experiments are similar to those of human experiments. In HIV patients, type II interferon is upregulated, and CD8+ T cells infiltrate in the lungs and exert major antiviral and bacterial functions [45].

ZNF683 mediates the transcription process in various types of tissue-resident lymphocytes, can promote lymphocyte retention in tissues [46]. Furthermore, ZNF683 is both necessary and sufficient for CD8+ T cells to produce IFNγ and promotes lymphocyte differentiation into long-lived effector lymphocytes in nonlymphoid organs and other non-barrier tissues, thus providing immediate immune protection against reinfection [29,47]. The transcriptome analysis results showed that ZNF683 was highly expressed in SPs. The results of real-time PCR were also consistent with these findings. Correspondingly, CD8+ T cell development and activation-related signaling pathways, including the T cell receptor signaling pathway, and proteins such as CD3E and CD8B were also enhanced in SPs, which may have been related to the upregulated expression of ZNF683. When we knocked down ZNF683 in human PBMCs and knocked out ZNF683 in NPMs PBMCs, the production of IFNγ and the proliferation of CD8+ T cells were inhibited. When ZNF683 was knocked down in human CD8+ T cells, the replication of HIV was increased. Due to the important role of ZNF683 in the immune system, we speculate that high expression of ZNF683 promotes CD8+ T cell IFNγ secretion and proliferation after infection to inhibit viral replication and slow the disease progression.

In addition, we briefly explored the mechanism of ZNF683 regulating IFNγ. As a transcription factor, ZNF683 can regulate gene expression and blimp-1 is a homologous gene of it [48]. Their gene structures are similar, and both have zinc finger domain. It has been reported that they can recognize the same site as transcription factors and inhibit gene expression. There is no IFNγ promoter in the site recognized by blimp [29,49]. We used AnimalTFDB to predict the transcription elements that can be bound by IFNγ promoter. As expected ZNF683 and blimp-1 were not captured, but other transcription elements that have been proved to be able to bind to IFNγ promoter were captured (Table S1), such as TBX21 (T-bet) [50], indicating that our prediction is reliable. To confirm it, we conducted a double luciferase reporter gene experiment and also found that overexpression of ZNF683 had no effect on the promoter activity of IFNγ (Figure S5). In addition, human ZNF683 and macaque ZNF683 are highly conserved and have the same zinc finger domain (Figure S6). We also did not capture ZNF683 in the prediction of transcription elements which can bound to macaque IFNγ promoter (Table S2), indicating that to increase IFNγ expression ZNF683 in macaque and human are not in a manner by directly binding to the promoter of IFNγ, the specific mechanism of ZNF683 regulating IFNγ secretion should be further explored.

In conclusion, 16S rDNA sequencing and transcriptome analysis were performed with tissue from the lungs of RPs and SPs in an SIV-infected macaque model. After the DEGs in the two groups were screened, ZNF683 expression was shown to be upregulated in SPs, and signaling pathways involved in the proliferation and activation of lymphocytes were also enhanced. Higher expression of ZNF683 promotes CD8+ T cell IFNγ secretion and proliferation after infection. IFNγ plays important roles in the control of virus replication and microbial invasion which can cause persistent inflammatory responses and lead to disease progression. These results indicate that ZNF683 may be a marker of CD8+ T cell function and associated with disease progression.

Unfortunately, there are many limitations and shortcomings of our research. First, we did not detect the dynamic indexes of these samples, so we could not determine the changes in the lung immune microenvironment during the whole infection process. Second, due to limited materials, the number of animals enrolled in this study is small and may not reflect the full characteristics after infection. In future research, we will perform more animal experiments and collect samples at different time points after infection to verify the function of ZNF683 in HIV infection.

## Figures and Tables

**Table 1:** Primers used to amplify related genes.

Target Gene	5′→3′
IFNγ-F	CTGTTACTGCCAGGACCCAT
IFNγ-R	TGCTACATCTGGATCACCTGC
IFNα-F	GCCTGAAGGACAGACATGACTTT
IFNα-R	GGATGGTTTGAGCCTTTTGG
GAPDH-F	GCT TGAGGGTTTGCTACAACATG
GAPDH-R	GACGCCTGCTTCACTACCTT
IFNβ-F	TGCCTCAAGGACAGGATGAAC
IFNβ-R	GCGTCCTCCTTCTGGAACTG
CD3E-F	GCCGCTTCTTCCTTTGAAGC
CD3E-R	ATCCAAGGGGGAGGGAATGA
CD8B-F	GCGTTCTGGTTTTGCTGGTT
CD8B-R	TGTAGTTTCCGTGCAGGCAT
ZNF683-F	CATATGTGGCAAGAGCTTTGG
ZNF683-R	AGAGCTTCACTCAACTTGCC-3
STAT1-F	TCTTCTGCCGGGTAGTTTCG
STAT1-R	CTCGAGGATGGCATACAGCA
IRF4-F	TGTGAAAATGGTTGCCAGGTG
IRF4-R	TCACGAGGATTTCCCGGTAG
IL1R2-F	TGCTCTTAAAAACTAGCCACGCA
IL1R2-R	CGTGGCAGAACCTGCTTTGA
CXCL8-F	ACTCCAAACCTTTCCACCCC
CXCL8-R	TTCCTTGGGGTCCAGACAGA
18S-F	GGACAACAAGCTCCGTGAAGA
18S-R	CAGAAGTGACGCAGCCCTCTA

## Data Availability

The data presented in this study are available in and NCBI SRA database (SRR9278630 to SRR9278631, SRR9278634 to SRR9278639, SRR9278643 to SRR9278644, and SRR9050947 to SRR9050956). All the original data are available from the corresponding author as required.

## Supplementary Materials

The following supporting information can be downloaded at: https://www.mdpi.com/article/10.3390/v14040719/s1, Figure S1: Bar graph of the LDA value distribution. The graph shows the species with LDA scores greater than the set value (default setting: 4.0). The length of each bar represents the influence of the species with distribution differences (RP/N: RP, *n* = 3; SP/S: SP, *n* = 3; healthy/H: SIV-, *n* = 4). Figure S2: HE staining of lungs. Figure S3: Overall distribution of differentially expressed genes (DEGs) in the two groups. (A) RP group. (B) SP group. Each point represents a gene. Green dots represent downregulated DEGs, red dots represent upregulated DEGs, and black dots represent non-DEGs. RP: *n* = 3; SP: *n* = 3; SIV-: *n* = 4. Figure S4: Expression of *ZNF683* in wild-type human PBMC, wild-type human CD8+ T cells, knockdown human PBMC, and knockdown human CD8+ T cells (A) and in wild-type macaque PBMC and knockout macaque PBMC. Figure S5: Effect of ZNF683 on IFNγ promoter. ZNF683 and promoter represent only transfection ZNF683 and IFNγ promoters, respectively. ZNF683-promoter represents co-transfection of ZNF683 and IFNγ promoter. Figure S6: Sequence alignment of *ZNF683* between human and macaque. Table S1: The gene which promoter binds to ZNF683 in human. Table S2: The gene which promoter binds to ZNF683 in macaque.
