# Supplementary material for: Transcription Factor ZNF683 Inhibits SIV/HIV Replication through Regulating IFNγ Secretion of CD8+ T Cells
Source: Viruses. 2022 Mar 30;14(4):719. doi: 10.3390/v14040719 (PMC9030044; doi:10.3390/v14040719)
Supplement: Supplementary file 1 [file viruses-14-00719-s001.zip › viruses-1618520-supplementary.pdf]

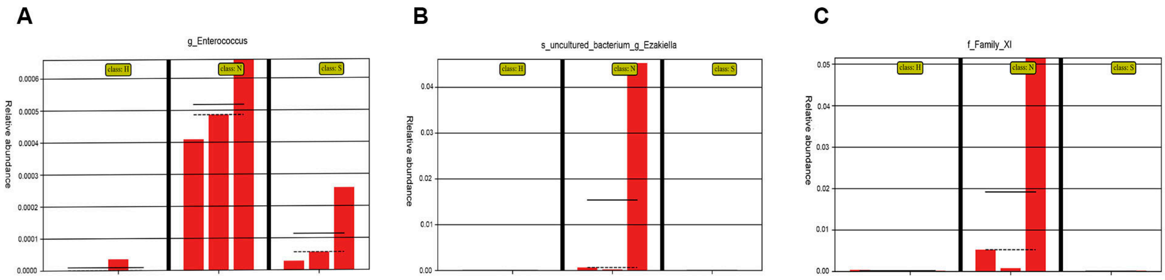

Figure S1. Bar graph of the LDA value distribution. The graph shows the species with LDA scores greater than the set value (default setting: 4.0). The length of each bar represents the influence of the species with distribution differences (RP/N: RP, n = 3; SP/S: SP, n = 3; healthy/H: SIV-, n = 4).

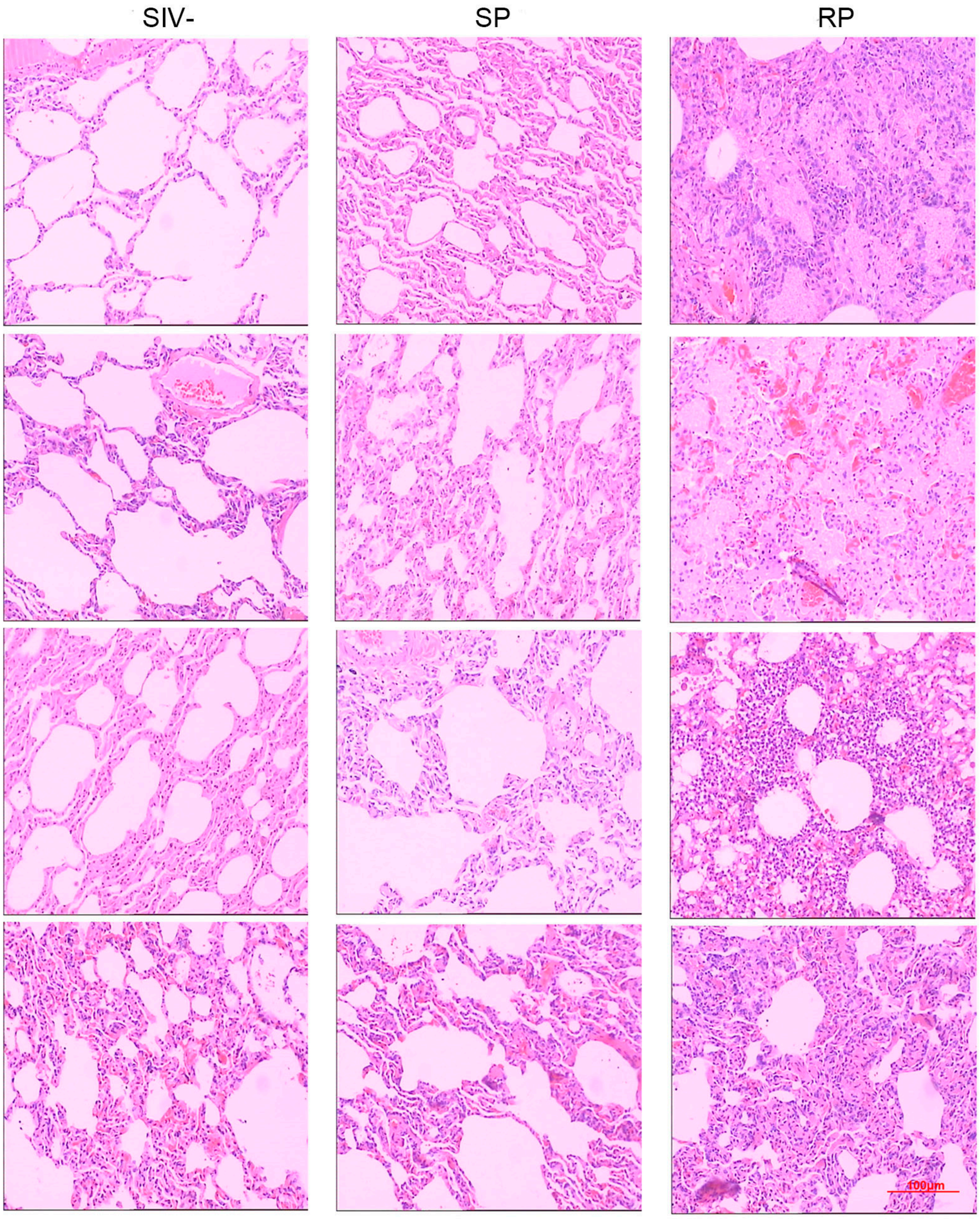

Figure S2. HE staining of lungs.

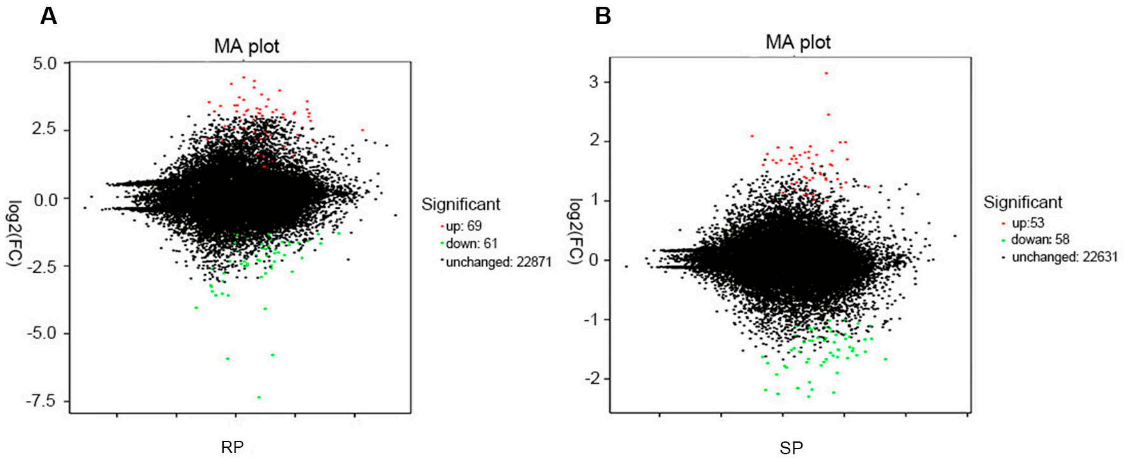

Figure S3. Overall distribution of differentially expressed genes (DEGs) in the two groups. (A) RP group. (B) SP group. Each point represents a gene. Green dots represent downregulated DEGs, red dots represent upregulated DEGs, and black dots represent non-DEGs. RP: n = 3; SP: n = 3; SIV-: n = 4.

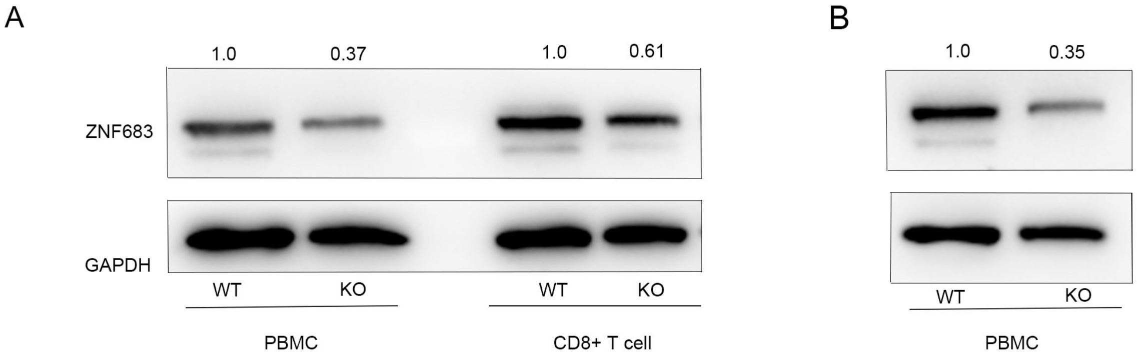

Figure S4. Expression of ZNF683 in wild-type human PBMC, wild-type human CD8+ T cells, knockdown human PBMC, and knockdown human CD8+ T cells (A) and in wild-type macaque PBMC and knockout macaque PBMC.

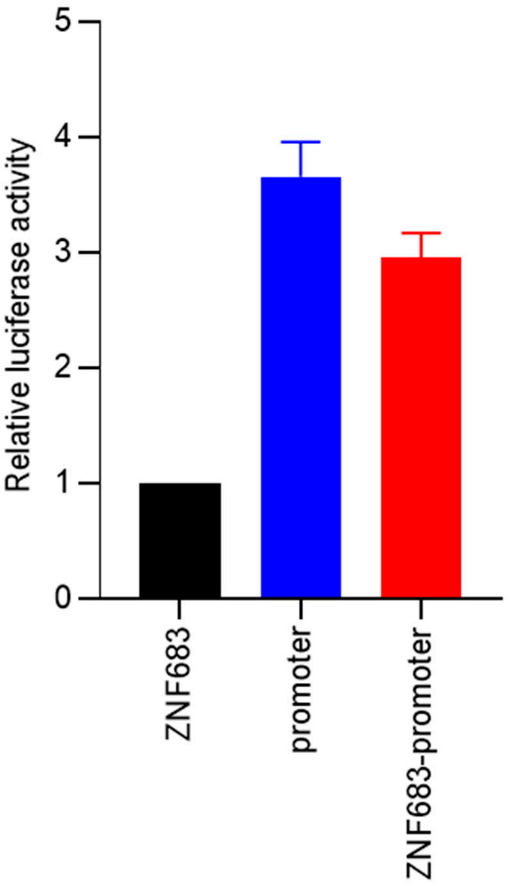

Figure S5. Effect of ZNF683 on IFN promoter. ZNF683 and promoter represent only transfection ZNF683 and IFN promoters, respectively. ZNF683-promoter represents co-transfection of ZNF683 and IFN promoter.

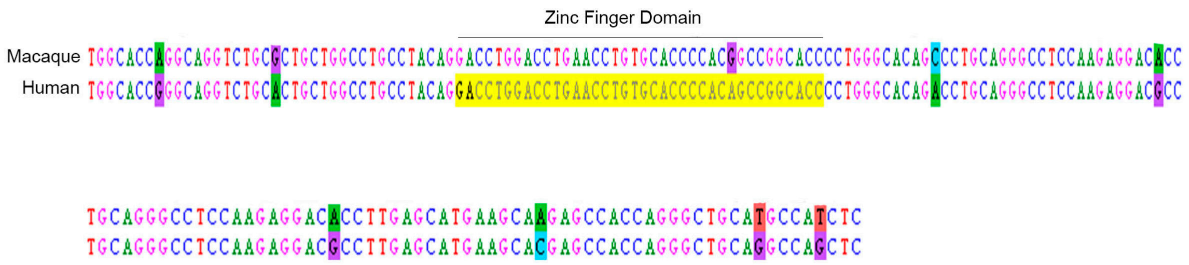

Figure S6. Sequence alignment of ZNF683 between human and macaque.
